# Acidosis promotes exon skipping through sequestering SR-rich splicing factors in nuclear speckles

**DOI:** 10.3389/fmolb.2026.1862962

**Published:** 2026-09-11

**Authors:** Alina S. Shtork, Pavel V. Rubtsov, Anastasia N. Kazakova, Iuliia I. Pavlova, Olga M. Ivanova, Sabina E. Illarionova, Margarita E. Bogomiakova, Svetlana V. Sizova, Galina M. Proshkina, Aleksandra A. Strokach, Ksenia M. Klimina, Victoria O. Shender, Sergey M. Deyev, Georgij P. Arapidi, Anna M. Varizhuk

**Affiliations:** 1 Lopukhin Federal Research and Clinical Center of Physical-Chemical Medicine of Federal Medical Biological Agency, Moscow, Russia; 2 Shemyakin-Ovchinnikov Institute of Bioorganic Chemistry of the Russian Academy of Sciences, Moscow, Russia

**Keywords:** acidosis, alternative splicing, hnRNP, nuclear speckles, SRSF

## Abstract

Deregulation of alternative splicing has long been associated with aggressive cancer phenotypes characterized by acute or chronic acidosis. Recently, it was hypothesized that this deregulation results from the sequestration of SR-rich splicing factors in nuclear speckles. However, this hypothesis lacked experimental evidence. Here, we demonstrate that, within the pH range consistent with acidosis, phosphorylated SR-rich fragments of splicing factors undergo a phase transition in minimal and multicomponent models of nuclear speckles. This acidification effect resembles the effect of SR dephosphorylation. We explain the molecular basis of this effect and visualize analogous transitions of nuclear speckles in acidified human cells. We also analyze splicing alterations in response to prolonged acidification. The most frequent alteration is exon skipping, consistent with the inaccessibility of SR-rich splicing factors, which normally promote exon inclusion. Our results support the previously proposed hypothesis and deepen the understanding of splicing regulation.

## 1 Introduction

Aberrant alternative splicing (AS) is now widely recognized as a hallmark of cancer, encouraging the search for its endogenous drivers and new therapeutic vulnerabilities in the splicing machinery (Bradley and Anczuków, 2023). Among metabolic factors presumed to cause AS dysregulation (Cui et al., 2024), acidosis stands out due to association with aggressive cancer phenotypes (Corbet and Feron, 2017; Pillai et al., 2019). However, the evidence for a specific impact of acidosis on AS rather than constitutive splicing is missing. AS suggests the dependence of splice site selection on the presence and accessibility of cis- and trans-regulatory elements (Tao et al., 2024). The former can be either exonic (EREs) or intronic (IREs). The latter include two major protein families: SR-rich splicing factors (SRSFs) and heterogenous nuclear ribonucleoproteins (hnRNPs). Members of both families contain at least one ERE/IRE-binding RNA recognition motif (RRM) and a conformationally disordered low complexity region (LCR).

The defining feature of SRSF is an SR-rich LCR, which supposedly binds to the pre-spliceosomal complex E and facilitates its transition to productive complexes A/B/C (Li et al., 2023). The binding sites of SRSF RRMs are enriched in exons (Pandit et al., 2013), and the recruitment of SRSFs to these sites promotes spliceosomal assembly at the nearby splice sites. Thus, major binding sites of SRSFs can be categorized as exonic splicing enhancers, and the upregulation of SRSF function is supposed to promote exon inclusion. LCRs of hnRNPs are typically rich in glycine, proline, or acidic amino acid residues and/or RGG motifs (Geuens et al., 2016). They can also interact with complex E but tend to hamper its transition to the productive complexes. The binding sites of hnRNP RRMs are enriched in introns, and the recruitment of hnRNPs to these sites prevents spliceosomal assembly (Huelga et al., 2012). Thus, major binding sites of hnRNPs can be categorized as intronic splicing silencers, and the upregulation of hnRNP function is supposed to promote exon skipping or intron retention.

Notably, there are nuances to SRSF and hnRNP activities. Both enhanced exon inclusion due to ERE-bound hnRNPs and repressed inclusion due to IRE-bound SRSF have been reported (Erkelenz et al., 2013). Those observations appear to contradict the above overview and could result from the intra-family competition between splicing factors and compensatory effects (Huelga et al., 2012; Pandit et al., 2013). Moreover, the above overview does not account for the roles of exonic splicing silencers or intronic splicing enhancers, the exon-independent recruitment of SRSFs (Jobbins et al., 2022), etc. Nevertheless, in most cases SRSFs and hnRNPs have the opposite functions, and the outcome of AS depends critically on their regulation by post-translational modifications (PTMs) and phase transitions–specifically, by liquid-liquid phase separation (LLPS). The most common PTM of SRSFs, critical for AS, is serine phosphorylation by SR-specific protein kinases (SRPKs) (Zhou and Fu, 2013), Cdc2-like kinases (CLKs) (Song et al., 2023), or casein kinase 2 (CK2) (Zhang et al., 2025). Phosphorylation activates SRSFs for binding complex E upon splicing (Saha and Ghosh, 2022), and subsequent dephosphorylation enables them to assist in mRNA export from the nucleus and translation initiation (Huang et al., 2004; Michlewski et al., 2008). Malfunction of SRPKs, CLKs, or CK2 has been implicated in oncogenic exon inclusion and deregulation of mRNA shuttling between the nucleoplasm and the cytoplasm (Naro et al., 2021).

Most of the time throughout the interphase, SRSFs are partially phosphorylated and localize to the nucleus, where they co-separate with hnRNPs, RNA, and small nuclear ribonucleoproteins (snRNPs) into relatively loose and therefore irregularly shaped biocondensates known as nuclear speckles (Galganski et al., 2017). These speckles are held together by transient homotypical or heterotypical LCR-LCR contacts (Martin et al., 2021; Zhang et al., 2024), RRM-RNA contacts, etc. The RNA components of the speckles include polyadenylated RNAs, small nuclear RNAs (snRNAs), and certain long non-coding RNAs (lncRNAs), of which MALAT is arguably the key one, as it contains multiple SRSF binding sites (Tripathi et al., 2010). Importantly, SRSFs scaffold the core of the speckles, while hnRNPs, spliceosomal components, and lncRNAs are enriched in the outer shell, and polyadenylated RNAs contact both core and peripheral speckle proteins (Paul et al., 2024). Factors affecting peripheral speckle components can cause the redistribution of speckles relative to chromatin (Alexander et al., 2025). Factors affecting core components can disrupt speckles or alter their morphology. One example is taurine, a metabolic modulator that inhibits SRSF-driven LLPS and induces speckle dissociation (Cui et al., 2024).

Acidosis is assumed to act oppositely to taurine, namely promote SRSF coarsening or solidification to reshape speckles (Cui et al., 2024). This assumption awaits verification and relies on the analogy between pH alteration and SRSF (de)phosphorylation, both of which alter net charge per residue (NCPR) and blockiness of charge (BLC) in SRSF LCRs.

In this study, we aimed to elucidate whether moderate acidification characteristic of metabolic disbalance in tumor microenvironment affects speckles and whether it resembles SRSF dephosphorylation. We verify the hypothesis of acidosis-induced speckle reshaping (Cui et al., 2024), clarify the role of phosphorylated SRSFs in this reshaping, touch upon its interference with hnRNP dynamics, and characterize its consequences with respect to AS.

## 2 Methods

### 2.1 Assembly of model speckles *in vitro*

#### 2.1.1 SR-rich peptides and other components of model speckles

The SR-rich peptide phospho-SR (PRS^p^PS^p^YGRS^p^RSRSRSRSRSR; >90% purity, according to LCMS data) with three phosphorylated residues (S^p^) was obtained from the Collective use center of Emanuel Institute of Biochemical Physics (Russia). The control peptides dephospho-SR (no phosphorylated residues) and mutant-SR (S/E mutations at phosphorylation sites), as well as their extended analogues dephospho-SR_ext (PRSPSYGRSRSRSRSRSRSRSRSNSRSRSYSP) and mutant-SR_ext (PREPEYGRERSRSRSRSRSRSRSNSRSRSYSP) were synthesized on a Liberty Blue Synthesizer (CEM corporation, Matthews, United States) following the Fmoc strategy and standard protocols for the solid-phase peptide synthesis with microwave treatment of the reaction mixture. Synthesis of the control peptides, as well as HPLC purification (final purity >90%), purity verification by LCMS, and labeling of all peptides with fluorescein isothiocyanate (FITC) were performed as described previously (Vedekhina et al., 2024). Mixed-sequence RNA from *Torula* yeast was obtained from HiMedia Laboratories (Thane, India). The full-length hnRNP A1 with a 6xHis tag was obtained from AMSBIO (Abingdon, United Kingdom) and labeled using the RED-Tris-NTA kit (NanoTemper Technologies, Germany) following the manufacturer’s protocol.

The net charge per residue (NCPR) of SR-rich peptides at different pH was calculated using the Prot pi online tool, version 2.2.29.152. BLC was calculated using the previously published equation (Yamazaki et al., 2022):

$$BCL=\left({\mathrm{MAX}}_{+}+{\mathrm{MAX}}_{-}\right)*\;\frac{\sum_{k=1}^{Nd}d_{k}\left(+,-\right)}{Nd\left(+,-\right)}/\left(\;\frac{\sum_{k=1}^{Nd}d_{k}\left(+,+\right)}{Nd\left(+,+\right)}+\frac{\sum_{k=1}^{Nd}d_{k}\left(-,-\right)}{Nd\left(-,-\right)}\right)$$

where *MAX*_*+*_ and *MAX*_*−*_ are absolute positive and negative values in the charge plot; *d*_*k*_(*+, −*), *d*_*k*_(*−, −*), and *d*_*k*_(*+, +*) are distances between the charged residues in the charge pairs; *N*_*d*_(*+, −*), *N*_*d*_(*−, −*), and *N*_*d*_(*+,+*) are numbers of these pairs.

#### 2.1.2 Assembly of minimal and multicomponent model speckles

To characterize RNA-dependent phase separation of SR-rich peptides and obtain minimal models of speckle cores, unlabeled phospho-SR or the control peptide (dephospho-SR or mutant-SR) was mixed with RNA to a final concentration of each component of 1 mg/mL in a 20 mM sodium phosphate (pH 7.4 or 6.0) or 20 mM sodium acetate (pH 4) buffer supplemented with 10% polyethylene glycol PEG-400. To obtain multicomponent speckle models, phospho- or dephospho-SR (95% unlabeled and 5% FITC-labeled; total final concentration: 1 mg/mL) was mixed hnRNP A1 (95% unlabeled and 5% RED-labeled; total final concentration: 6 μM) and RNA (3 mg/mL) in 20 mM sodium phosphate buffer (pH 7.0 or pH 6.2) supplemented with 140 mM KCl and 10% PEG-400. This is a modified version of the previously published procedure (Vedekhina et al., 2024), in which a HEPES-based buffer was used instead of the sodium phosphate one used herein. The buffer was altered to shift the pH range so that it covers the moderately acidic medium.

### 2.2 Characterization of phase separation *in vitro*

#### 2.2.1 Turbidimetry and light microscopy

Efficiency of phase separation in SR-RNA mixtures was initially characterized by turbidimetry. The mixtures were prepared in Corning Flat Bottom Transparent Polystyrene plates (3 repeats on different days, each in duplicate), and absorbance at 600 nm was registered using Infinite 200 PRO plate reader (Tecan, MȨnnedorf, Switzerland). To avoid artifacts in aggregation-prone samples, the analysis was performed within 15 min after mixing with 30 s shaking every 5 min and immediately before measurements. For microscopy imaging, the samples were placed into the working chamber between the glass sides 5 min after mixing and left for additional 10–15 min to allow for the precipitation of the dense phase (droplets) on the bottom slide. The images were acquired using Eclipse Ti2 microscope (Nikon, Tokyo, Japan). The experiments were performed in triplicate, and the resulting images were used to calculate droplet fraction using DropletCalc software (Shtork et al., 2025).

#### 2.2.2 Fluorescent microscopy and fluorescence recovery after photobleaching (FRAP)

Phase separation in SR-RNA-hnRNP A1 mixtures containing FITC-labeled SR peptides and RED-labeled hnRNP A1 was characterized by fluorescence microscopy imaging using Eclipse Ti2 microscope (Nikon, Tokyo, Japan). FITC-SR peptides were visualized upon fluorescence excitation at 488 nm with a cut-off filter of 520 nm, and RED-hnRNP A1 was visualized upon excitation at 647 nm with a cut-off filter of 660 nm. The images were processed in ImageJ (version 2024) to evaluate the colocalization between FITC-SR-positive and RED-hnRNP-positive droplets and calculate the partitioning coefficients of the SR peptides and hnRNP A1. Droplet areas and circularity were assessed using DropletCalc software (Shtork et al., 2025). For each sample, six images from two biological repeats, three replicates each, were processed.

FRAP assays were performed using an FV3000 laser scanning confocal microscope (Olympus, Tokyo, Japan). In each assay, a fragment of the droplet was bleached using a 10× laser (10% intensity, 40 s). After that, time lapse images were acquired every 11 s at 1% laser intensity with λ_ex_/λ_em_ = 488/520–540 nm (FITC-SR) or 647/660–680 nm (RED-hnRNP A1). The fluorescence intensity of the bleached area was then normalized by the intensity of the nearest non-bleached droplet. The experiments were performed in three replicates. The resulting recovery curves were averaged and fitted to a mono-exponential equation.

#### 2.2.3 Passive microrheology

To partially verify the liquid state of minimal speckle models (SR-RNA droplets), droplet fusion on Tween-coated glass slides was monitored using Eclipse Ti2 microscope (Nikon, Tokyo, Japan). The slides and coverslips were coated with Tween20 (2% solution, v/v) as described previously (Alshareedah et al., 2021). To partially verify the liquid state of model speckles assembled from phospho-SR (1 mg/mL), hnRNP A1 (6 μM) and RNA (3 mg/mL) in 10% PEG-containing sodium phosphate buffer, pH 7.2 or 6.0, single particle tracking was used. The particles were 330 nm poly (acrolein-co-styrene) microspheres (Ac-St) loaded with 6 nm diameter CdSe/ZnS quantum dots (QD), which exhibited an emission maximum at 610 nm. QDs were a kind gift of Prof. Mikhail V. Artemyev from Belarusian State University.

The Ac-St microspheres were synthesized by surfactant-free radical copolymerization of acrolein and styrene at a monomer-to-water volume ratio of 1:9, using potassium persulfate (1.5 wt% relative to total monomer mass) as the initiator. The mixture was heated to 65 °C and polymerized under nitrogen atmosphere with continuous stirring for 12 h, following a previously reported protocol with minor modifications (Generalova et al., 2011). For QD loading, 0.2 mL of a 5 wt% aqueous suspension of polymer microspheres was mixed with 2-propanol, sonicated three times for 2 min each, centrifuged for 5 min, and redispersed in fresh 2-propanol. Particle swelling was induced by adding 0.2 mL of chloroform and incubating at room temperature for 1 h. Separately, 2 mg of QDs were purified from trioctylphosphine excess by repeated dispersion in chloroform and precipitation with methanol (1:3 v/v), dissolved in 2-propanol, and added to the polymer particle suspension (2-propanol/chloroform, 10:1 v/v). The mixture was vigorously stirred, sonicated for 2 min, and incubated for 20 min under stirring; this cycle was repeated three times, followed by shaking at room temperature for 2 h. Unbound QDs were removed by centrifugation at 10,000 rpm for 10 min and five washes with water. The labeled Ac-St microspheres were finally redispersed in 0.2 mL of water (Generalova et al., 2011). Particle size distribution was evaluated by dynamic light scattering using a 90 Plus Particle Size Analyzer (multimodal mode, 25 °C, 661 nm laser), yielding a hydrodynamic diameter D = 330 nm and polydispersity PD = 0.102%. The suspension of QD-loaded Ac-St spheres (0.5 wt%) was centrifuged briefly to discard occasional aggregates. Then, 1 μL of the suspension was added to 10 μL of the preassembled droplet sample, and the mixture was incubated for 5 min at room temperature, yielding 0-4 QD-loaded Ac-St microspheres per droplet, which was visualized using an FV3000 laser scanning confocal microscope (Olympus, Tokyo, Japan). The luminescence of the microspheres was excited by green light (488–520 nm) and visualized in a Cy3 channel (560–640 nm). The droplets containing single microspheres were selected for tracking using time lapse experiments. For higher time resolution, the tracking was repeated using Eclipse Ti2 microscope (Nikon, Tokyo, Japan). The video files were processed to extract the dependence of the microsphere mean square displacement (MSD) as a function of the lag time using custom software based on Python library for particle tracking TrackPy. The source code for this software is available at https://github.com/biopolymers-lab-FRCC-PCM/BrownianTrack. For each condition (pH 7.2 or pH 6.0), 150 tracks were processed. Linear fitting of the resulting average MSD plot provided the apparent diffusion coefficients (D = MSD/4tau).

### 2.3 Modeling acute acidosis in cell cultures and analysis of its impact of nuclear speckles

#### 2.3.1 Short-term acidification and pH imaging in the nucleus

Immortalized human embryonic kidney cells HEK-293 and neuroblastoma cells SH-SY5Y were cultured in a humidified 5% CO_2_ incubator at 37 °C, tested for *mycoplasma* contamination, and grown in 8-well glass slides. HEK-293 cells were maintained in Dulbecco’s Modified Eagle Medium (DMEM) (Paneco, Moscow, Russia), while SH-SY5Y cells were maintained in DMEM/F12 (Paneco, Moscow, Russia). Both media were supplemented with a 1% penicillin/streptomycin mixture (Paneco, Moscow, Russia), 2 mM glutamine (Paneco, Moscow, Russia), and 10% fetal bovine serum (Capricorn Scientific, United States). Cells were used at approximately 70% confluency. Then, the cells were transfected with the nucleotropic pH sensor FAM-C_5_T^propyl^-TAMRA using Lipofectamine3000 (Thermo Fisher Scientific, Waltham, MA, United States) following the manufacturer’s protocol, adjusted for a final extracellular sensor concentration of 150 nM, as in previous studies (Turaev et al., 2021; Petrunina et al., 2023).

Two hours after transfection, a cyclin-dependent kinase 1 (CDK1) inhibitor RO-3306 (MilliporeSigma, Burlington, MA, United States) was added to a final concentration of 8 μg/mL, and the cells were incubated for 18 h for synchronization in the G2 phase. Then, the cells were washed with PBS (20 mM sodium phosphate buffer containing 140 mM KCl), and incubated for 30 min in PBS, pH 7.4 (control) or pH 6.0 (acidosis), supplemented with valinomycin and nigericin (10 μM each). All buffers were supplemented with RO-3306 (8 μg/mL) to avoid cell cycle progression. After 30 min of incubation, the cells were fixed with 4% paraformaldehyde (PFA), and cell nuclei were stained with 1 μg/mL DAPI (Thermo Fisher Scientific, Waltham, MA, United States).

Fluorescence microscopy imaging of acidified and non-acidified cells transfected with FAM-C_5_T^propyl^-TAMRA was performed using a LSM 780 confocal laser scanning microscope (Carl Zeiss, Germany) with λ_ex_/λ_em_ = 405/450–480 nm (DAPI), 488/520–540 nm (FAM), or 550/580–680 nm (TAMRA). Acidification was confirmed by comparing the intensities of FAM signal in the nucleoplasm and sensor-stained speckles of the cells incubated with the physiological versus acidic buffer. TAMRA signal, which shows minimal pH-dependence within FAM-C_5_T^propyl^-TAMRA, was used as a reference.

#### 2.3.2 Immunostaining and morphological analysis of speckles

The initial analysis of the morphologies of HEK-293 and SH-SY5Y speckles stained with FAM-C_5_T^propyl^-TAMRA was performed based on TAMRA imaging. The results were further verified in HEK-293 cells by immunocytochemistry. For this, the cells were synchronized and incubated in native/acidic media with ionophores as described above (transfection with the sensor was skipped). After that, the cells were fixed with 4% PFA (15 min incubation), permeabilized with 0.1% Triton X-100 (10 min incubation), washed with Dulbecco’s phosphate-buffered saline (DPBS) and blocked with 4% BSA in DPBS for 1 h. To stain speckle components, the cells were incubated for 12 h with FITC-labeled anti-SRRM2/SRSF2 Ab, 5 μg/mL (#sc-53518, Santa Cruz Biotechnology, Dallas, TX, United States) or rabbit monoclonal anti-SF3B1Ab, 1/100 (#ab170854 Abcam, Cambridge, United Kingdom) in DPBS containing 4% BSA. To visualize the unlabeled anti-SF3B1 antibody, this step was followed by an incubation with Alexa Fluor 647-labeled goat anti-rabbit secondary antibody (CF647 20809-500, Biotium, Fremont, CA, United States). Cell nuclei were stained with DAPI (1 μg/mL), and the images were acquired using Eclipse Ti2 microscope (Nikon, Tokyo, Japan).

The morphologies of the speckles were characterized similar to those of model droplets, i.e., using median speckle area and circularity as the key parameters. Both parameters were calculated by processing fluorescence microscopy images in DropletCalc software. All experiments were performed in two biological repeats, each in three replicates. In each replicate, one to three images were obtained, which resulted in the following sample size (number or analyzed speckles): 148–152 (staining with FAM-C_5_T^propyl^-TAMRA), 52-55 (staining with anti-SRRM2/SRSF2 Ab), and 60-68 (staining with anti-SF3B1 Ab).

### 2.4 Modeling mild acidosis and analysis of its impact on transcription and splicing

#### 2.4.1 Prolonged acidification, RNA extraction, RT-PCR, and sequencing

HEK-293 cells were grown in 6-well plates in DMEM, supplemented with a 1% penicillin/streptomycin mixture, 2 mM glutamine, and 10% fetal bovine serum, to a confluency of 60%–70% and then incubated for 48 h under following conditions:

- DMEM, pH 7.4, 0.2% DMSO (control);
- DMEM, pH 6.6, 0.2% DMSO (acidosis);
- DMEM, pH 7.4, 10 μM SRPIN340 (MedChemExpress, Monmouth Junction, NJ, United States), and 0.2% DMSO (SRPIN treatment).

Acidic DMEM was obtained by titration of commercial DMEM (Paneco, Moscow, Russia) with HCl.

After 48 h incubation, the cells were harvested. Total RNA was isolated using RNeasy Mini Kit (Qiagen, Hilden, Germany) following the manufacturer’s instructions including the DNase treatment step. The quantity of the resulting RNA was evaluated and the purity was confirmed by measuring absorption at 260 nm/280 nm using NanoQuant plates and plate reader Infinite 200 PRO (Tecan, Männedorf, Switzerland). The experiments were performed in three biological repeats.

For quantitative analysis of relative gene expression by reverse transcription with PCR (qRT-PCR), complementary DNA (cDNA) was synthesized from 2 to 3 μg of isolated RNA per sample using Magnus reverse transcriptase (Evrogen, Moscow, Russia) and 40 pmol of random decamer primer (Evrogen, Moscow, Russia) following the manufacturer’s instructions.

Quantitative PCR was performed using qPCRmix-HS SYBR (Evrogen, Moscow, Russia). For each reaction, 1.5 μL of the cDNA was mixed with 5 pmol of the respective primers (Supplementary Table S1) and 5 μL of qPCRmix-HS SYBR. Then, RNase-free water was added to a total volume of 25 μL, and PCR was carried out in 45 cycles (95 °C for 3 min, then 95 °C for 10 s, 60 °C for 20 s, and 72 °C for 30 s per cycle) using CFX96 Touch RT PCR Detection System (Bio-Rad, Hercules, CA, United States). The experiments were performed in two series: six genes/transcript variants of interest and a reference gene (*TBP*) were compared in each series, all in three replicates for conditions a-c (control, acidosis, and SRPIN). Relative expression was calculated using the ΔΔCq method: Ratio^acidosis/SRPIN^ = 2^(−ΔCq^acidosis/SRPIN^+ΔCq^control^); ΔCq = Cq^gene of interest^ – Cq^*TBP*^. To evaluate the inclusion of a differentially skipped exon, the adjacent constitutive exon was used as a reference, and the reference-skipped exon junction was the cDNA of interest. Ratio^junction^ = 2^(Cq^reference^ − Cq^junction^). Under each condition (control/acidosis/SRPIN), RT-PCR data from three biological repeats were processed independently.

For RNA-sequencing (RNA-seq), 300–400 ng total RNA was used. Sequencing was performed for two biological repeats (three for acidosis), each in three replicates. Libraries were prepared using KAPA mRNA Capture Kit (Roche, Bazel, Switzerland) and KAPA RNA HyperPrep Kit (Roche, Bazel, Switzerland). Libraries were purified using KAPA HyperPure Beads (Roche, Bazel, Switzerland), and size distribution and quality were assessed using a High Sensitivity DNA chip (Agilent Technologies, Santa Clara, CA, United States). Libraries were quantified using the Quant-iT DNA Assay Kit, High Sensitivity (Thermo Fisher Scientific, Waltham, MA, United States). Sequencing was performed on the NextSeq 1000 (Illumina, San Diego, CA, United States) using the NextSeq 1000/2000 P2 Reagents (200 Cycles), with 2% PhiX (Illumina) added as an internal control.

#### 2.4.2 Analysis of gene expression and alternative splicing

To improve read quality prior to mapping, raw paired-end reads were trimmed using Trimmomatic (v. 0.39) and Trimgalore (v. 0.6.6) (Bolger et al., 2014). Transcript-level abundances were quantified for each sample using a quasi-mapping approach implemented in Salmon (v. 1.5.1) (Patro et al., 2017), with the Gencode GRCh38.p13 reference transcriptome and default parameters. Then transcript-level abundances were aggregated to the gene-level counts using the Tximport R package (Soneson et al., 2016). Differential expression analysis was performed with DESeq2 (Love et al., 2014). False discovery rate (FDR) was controlled using the Benjamini–Hochberg approach. Genes were considered lowly expressed and excluded from further analysis if the total TPM across samples was below predefined threshold of 6 TPM in both the control and treatment groups.

For alternative splicing analysis, trimmed paired-end reads were mapped to the Gencode GRCh38.p13 reference genome using STAR (v. 2.7.9a) (Dobin et al., 2013) with the following parameters: maximum number of multiple alignments per read was 10; non-canonical junctions were removed; the minimum splice overhang was eight for unannotated junctions and one for annotated junctions; the mismatch rate was limited to 6% of the read length; minimum intron length was 20 bp and maximum intron length was 1,000,000 bp; a two-pass mode was enabled. Splicing events were quantified using rMATS (Shen et al., 2014) with the following parameters recommended by the developers: -cstat 0.0001 and -novelSS. Alternative splicing (AS) events were considered significant at FDR <0.05 and IncLevelDifference >5%.

Functional gene annotation was conducted using the Reactome and Gene Ontology (GO) databases. Pathway enrichment analysis was performed using the clusterProfiler (Yu et al., 2012), ReactomePA (Yu and He, 2015) and msigdbr (Liberzon et al., 2011) packages. Frequencies of SRSF binding motifs in differentially skipped exons were calculated using the ESE finder tool, version 3.0 (Cartegni, 2003) with a motif score threshold of 2.

## 3 Results

### 3.1 Acidification induces phase separation of a phosphorylated SRSF fragment in the presence of RNA

We assumed that phosphorylated SR-rich LCRs account for the sensitivity of SRSFs to acidification within the biologically relevant pH range of 6.0–7.4, which covers extracellular and intracellular pH values associated with cancer and inflammation (Damaghi et al., 2013; Okajima, 2013; Korenchan and Flavell, 2019). Side chains of unmodified amino acids, excluding His (pKa 6.0), have pKa values that are well outside this range. In contrast, the side chain of phosphoserine (S^p^) has a pKa of 5.8 for the second proton (Śmiechowski, 2010), suggesting substantial changes in the net charge per residue (NCPR) of S^p^-containing sequences between pH 6.0 and 7.4. These changes may be accompanied by alterations in blockiness of charge (BLC) and LLPS potential.

**FIGURE 1:**
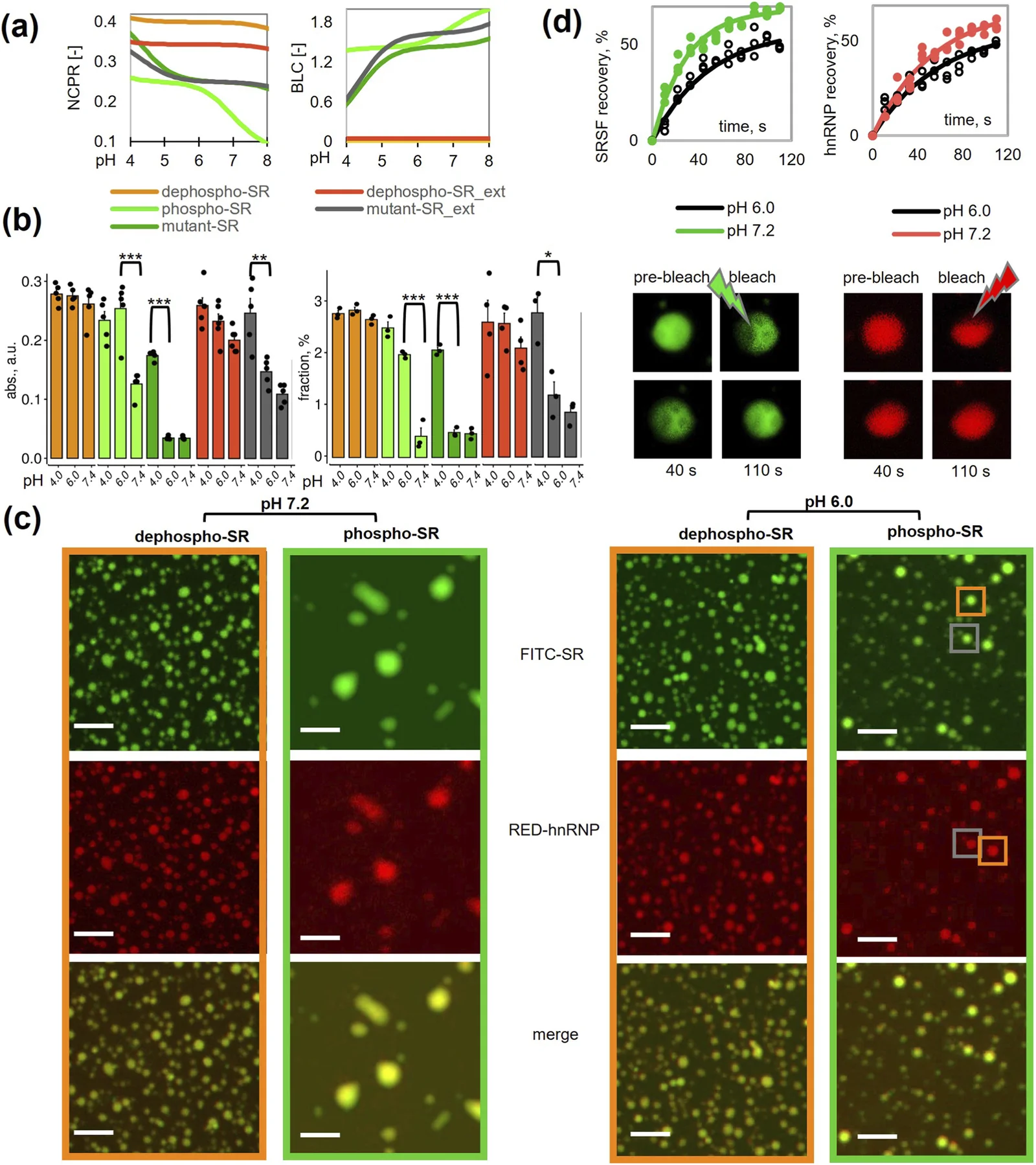
Phase-separation of the (de)phosphorylated SRSF fragment (SR) in different media and its co-separation with hnRNP A1. **(a)** Predicted pH-dependence of the separation-defining parameters of the SR variants, average net charge per residue (NCPR) and blockiness of charge (BLC). **(b)** Quantitative analysis of the relative separation efficiency by turbidimetry (absorbance at 600 nm, left graph) and microscopy (droplet fraction, right). Conditions: 1 mg/mL SR and 1 mg/mL RNA in a 20 mM sodium phosphate (pH 7.4 or 6.0) or 20 mM sodium acetate (pH 4) buffer supplemented with 10% PEG. **p < 0.01; ***p < 0.001. **(c)** Co-separation of (de)phospho-SR (green) and hnRNP A1 (red). Scale bar: 10 μm. Conditions: 1 mg/mL (de)phospho-SR (5% FITC-labeled), 6 μM hnRNP A1 (5% RED-labeled), and 3 mg/mL RNA in 20 mM sodium phosphate buffer supplemented with 140 mM KCl and 10% PEG-400. **(d)** Fluorescence recovery after photobleaching of phospho-SR-hnRNP A1 droplets at pH 6 versus pH 7 (top) and examples of selected bleached droplets pH 6 at different time points. In the large-field image, the droplets selected for bleaching and signal normalization are in orange and grey boxes, respectively.

To verify these assumptions, we selected an SRSF1 fragment containing experimentally confirmed phosphorylation sites in the N-terminal region (aa 197–217) (Zhou et al., 2013; Maertens et al., 2014) as a representative SR-rich LCR. Its triple-phosphorylated variant (PRS^p^PS^p^YGRS^p^RSRSRSRSRSR) is referred to hereafter as phospho-SR. The unphosphorylated variant PRSPSYGRSRSRSRSRSRSR and its extended analog PRSPSYGRSRSRSRSRSRSRSRSNSRSRSYSP (aa 197–229) were used as a dephospho-SR and dephospho-SR-ext controls. Additional control peptides were mutant-SR (PREPEYGRERSRSRSRSRSR) and its extended analog mutant-SR_ext (PREPEYGRERSRSRSRSRSRSRSNSRSRSYSP). They had decreased positive NCPR and increased BLC compared to dephospho-SR, almost similar to those of phospho-SR at pH 6, due to three S→E substitutions. Such mutations are frequent in eukaryotes and are commonly acknowledged as phosphomimic (Pearlman et al., 2011). However, unlike S^p^, the glutamic acid side chain (pKa 4.1) is deprotonated throughout the biologically relevant pH range (6.0–7.4). Thus, unlike phospho-SR, mutant-SR is expected to exhibit consistent properties within this pH range. The NCPR and BCL profiles of dephospho-, phospho-, and mutant-SR predicted based on the pKa values of R, Sp, and E are shown in Figure 1a. We tested whether the inflection points in these profiles are in line with changes in phase separation efficiency.

We analyzed phase separation of dephospho-/phospho-/mutant-SR peptides in the presence of heterogenous (3–40 kDa) mixed-sequence RNA from *Torula* yeast, which, in the first approximation, mimics heterogenous RNA in nuclear speckles. In the absence of RNA, the SR peptides formed occasional irregular aggregates in acidic media and were mostly soluble at pH 7.4 (Supplementary Figure S1). In the presence of RNA, phase separation became reproducible when biopolymer charges nearly compensated each other, i.e., at 1:1 mass equivalent ratio (Supplementary Figure S1). A further increase in RNA concentration, up to 1.5 mass equivalents, had no apparent effect; thus, all subsequent quantitative analyses of phase separation were performed using 1:1 SR-RNA mixtures. The dense phase consisted of micrometer-size droplets (Supplementary Figure S1) that scattered light and precipitated slowly. This enabled the monitoring of phase separation using turbidimetry within 15 min after mixing, as well as the quantification of the dense phase fraction using microscopy-based analysis of the total area of droplet projections on glass slides (Figure 1b). It should be noted that the projection area calculations were the least accurate for phospho-SR due to the interference of the unsettled (floating) droplets. The residual floating was less pronounced for dephospho-SR, which enabled us to obtain high-contrast images and track occasional fusion of the smaller droplets (Supplementary Figure S2 and Supplementary Data Sheet 1), in line with their presumed liquid state.

For all SR variants, the separation efficiency was the highest at pH 4.0 and the lowest at pH 7.4, according to both turbidimetry and microscopy (Figure 1b). Phospho-SR showed minor separation, comparable to that of mutant-SR (_ext), at pH 7.4, while at pH 6.0 it behaved similar to dephospho-SR (_ext). The peptides exhibited similar trends in their minimal (dephospho/mutant-SR) and extended (dephospho/mutant-SR_ext) variants. Mutant-SR (_ext) resembled dephospho-SR (_ext) only at pH 4.0 but not at pH 6.0. These results agree with NCPR and BLC profiles (Figure 1a) and suggest a phase transition of mutant-SR (_ext) at pH < 6.0, a transition of phospho-SR between pH 6.0 and 7.4, and efficient pH-independent separation of dephospho-SR (_ext) with no apparent transitions between pH 4.0 and pH 7.4 (Figure 1b).

We conclude that minor acidification promotes phase separation in phospho-SR and RNA-containing solutions and may have similar effects on full-size SRSFs in the presence of RNA in the core of the nuclear speckles. Next, we investigated whether proteins from the speckle periphery (hnRNPs) could engage in this process. We performed additional phase separation assays with (de)phospho-SR as a core component and the full-length hnRNP A1 as a typical periphery component.

### 3.2 Acidification alters co-separation of phospho-SRSF with hnRNP A1 and reshapes nuclear speckles

The co-separation of hnRNP A1 with (de)phospho-SR in the presence of RNA was studied using fluorescence microscopy with terminally labeled SR and hnRNP A1 in nearly neutral (pH 7.2) and mildly acidic (pH 6.0) media. Phospho-SR and dephospho-SR were covalently labeled with fluorescein isothiocyanate (FITC), and His-tagged hnRNP A1 was noncovalently labeled with a far-red light-emitting dye (RED). Representative images of the SR-hnRNP-RNA mixtures are shown in Figure 1c. Additional large-field images are presented in Supplementary Figure S3.

hnRNP A1 co-separated efficiently with dephospho-SR, yielding regular-shaped droplets with a median circularity of ∼0.81 and an average diameter of ∼1.0 μm at pH 6.0 and 7.2. Colocalization between the RED-hnRNP and FITC-SR signals exceeded 90%. Partitioning coefficients were similar at pH 6.0 and pH 7.2 (7 ± 1 for hnRNP A1 and 13 ± 2 for dephospho-SR). In contrast, the partitioning coefficient of phospho-SR was significantly lower at pH 7 (5.2 ± 0.1) than at pH 6 (12 ± 1). The co-separation of hnRNP A1 with phospho-SR was also efficient (Figure 1c), but the median circularity of the droplets decreased slightly (from ∼0.81 to ∼0.79) at pH 7.2 (Supplementary Figure S3). Since diameter is an inadequate parameter for characterizing irregularly shaped droplets, projection area was used instead. The median values at pH 6.0 (approximately 3.0 μm^2^) were similar for phospho- and dephospho-SR. However, at pH 7.2, the median value increased slightly (to approximately 3.5 μm^2^) for phospho-SR. We conclude that acidification made the phospho-SR-hnRNP A1 droplets more compact and presumably more viscous.

Such changes suggest altered mobility of the components, which was verified using fluorescence recovery after photobleaching (FRAP). The recovery curves are shown in Figure 1d. Representative images of the partially bleached droplets at pH 6.0 and pH 7.2 are shown in Figure 1d and Supplementary Figure S3, respectively. In neutral media, partial (up to ∼70%) FRAP of phospho-SR was nearly twice as fast as that of hnRNP A1 (tau^1/2^_SR_ = 20 ± 1 s versus tau^1/2^_hnRNP_ = 37 ± 2 s), indicating rapid diffusion of phospho-SR, which is consistent with its low molecular weight compared to the full-size hnRNP. Acidification increased intra-droplet fluorescence but decreased its normalized recovery. The effect of acidification on the kinetics of hnRNP A1 FRAP was minor, whereas phospho-SR showed a significantly slower FRAP (tau^1/2^_SR_ increased to a value of 34 ± 1 s). To summarize this part, FRAP assays confirmed the mobility of co-separated phospho-SR and hnRNP A1, consistent with the presumed liquid state of the condensates. The mobility of both components, especially phospho-SRSF, was reduced at pH 6.0 compared to pH 7.2, pointing to more viscous droplets in acidic media.

The motion of any inert particle should be reduced in viscous droplets, so we used intra-droplet tracking of polymeric microspheres to complement FRAP data and additionally verify the pH effects. The microspheres were obtained by copolymerization of acrolein and styrene and loaded with red light-emitting CdSe/ZnS quantum dots (QD) for fluorescence imaging (Generalova et al., 2011). Analysis of their size distribution by dynamic light scattering (Supplementary Figure S4a) revealed low polydispersity and an average size of 330 nm (Supplementary Figure S4b). The microspheres entered the droplets (Supplementary Figure S5b), and the motion of single spheres was tracked by fluorescence microscopy (Supplementary Data Sheet 2). The resulting trajectories were consistent with Brownian motion at both pH 7.2 and 6.0. However, analysis of mean square displacements (fitting their dependence on the lag time to a linear function) revealed significantly slower diffusion at pH 6.0 compared to 7.2 (Supplementary Figure S4c): D = (0.58 ± 0.02)*10^−3^μm^2^s^−1^ and (1.2 ± 0.7)*10^−3^ μm^2^s^−1^, respectively. These results support increased droplet viscosity in acidic media.

Next, we verified analogous reshaping of nuclear speckles upon acute acidosis. To model this, human embryonic kidney cells (HEK-293) were treated with an acidic buffer (pH 6) supplemented with ionophores. Prior to acidification, the cells were synchronized in the G2 phase using the cyclin-dependent kinase (CDK) inhibitor RO-3306 (Vassilev, 2006) to minimize cycle-dependent variance in speckle morphology. To ensure acidification reaches the nucleus, cells were transfected with a nucleotropic pH sensor FAM-C_5_T^propyl^-TAMRA (Petrunina et al., 2023). This ratiometric sensor is based on a cytosine-rich oligonucleotide labeled with 6-caroxyfluorescein (FAM) and tetramethylrhodamine (TAMRA) residues and reportedly stains both the speckles and the nucleoplasm. In acidic media, the oligonucleotide core adopts a noncanonical secondary structure that quenches FAM fluorescence, thus decreasing the FAM:TAMRA signal ratio. The sensor was chosen because of its sharp pH dependence between pH 6.0 and 7.4, with a transition point of 7.0 ± 0.1 in lung adenocarcinoma cells (Petrunina et al., 2023).

**FIGURE 2:**
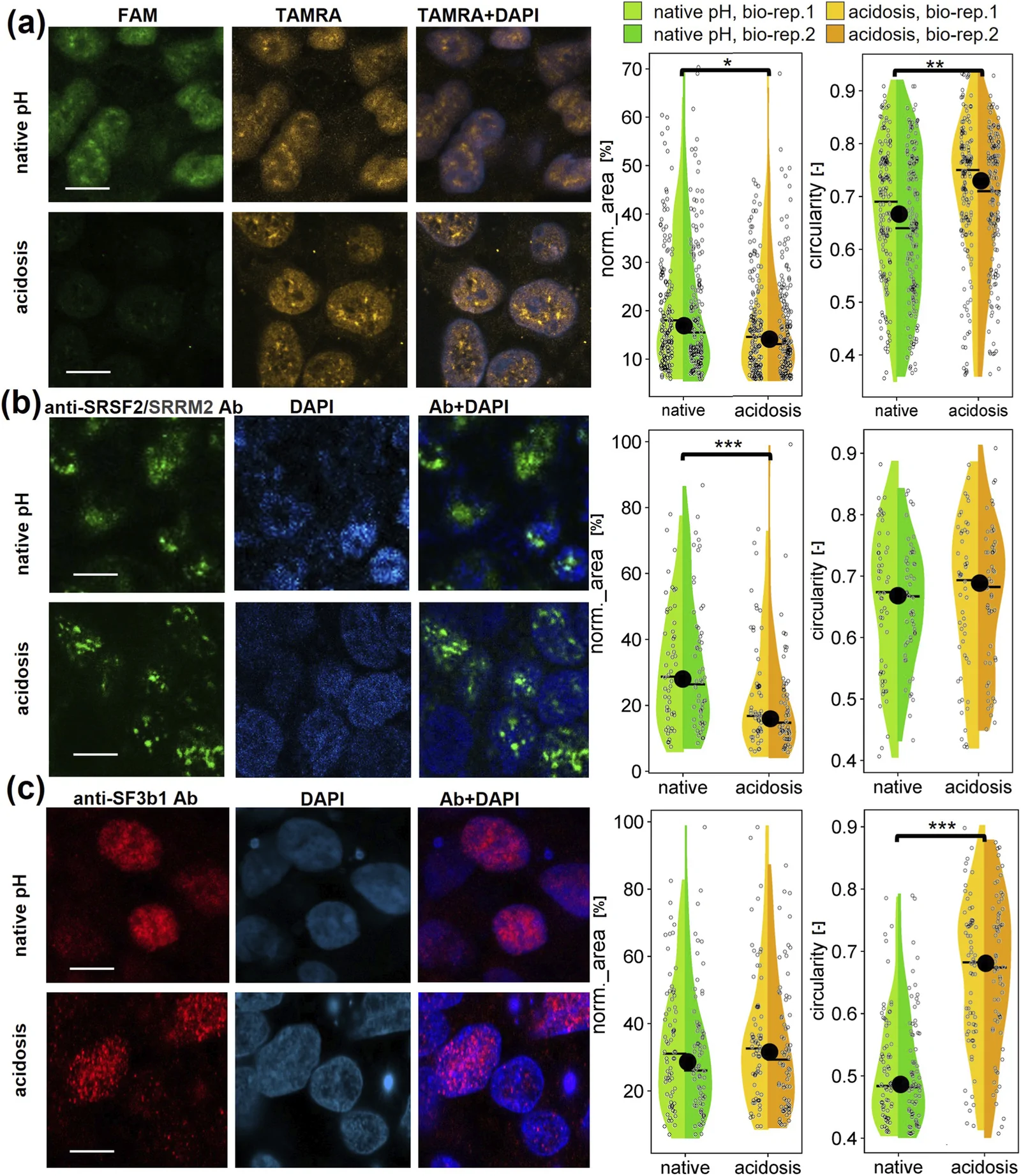
Morphologies of nuclear speckles in G2-synchronized HEK-293T cells after incubation in native or acidified media with ionophores. **(a)** Representative fluorescence microscopy images of HEK-293 cells transfected with the nucleotropic ratiometric pH sensor FAM-C5T^propyl^-TAMRA (left) and summary of the TAMRA foci morphologies (right). Norm._area [%] is the area of a TAMRA focus normalized by a maximum value for both groups (1.2 μ^2^). Sample size: 148–152 foci for each biological repeat. **(b)** Representative images of the cells stained with FITC-labeled anti-SRRM2/SRSF2 primary antibody (left) and summary of the SRRM2/SRSF2 foci morphologies (right). Norm._area [%] is the area of a SRRM2/SRSF2 focus normalized by a maximum value for both groups (1.7 μ^2^). Sample size: 52–55 foci for each biological repeat. **(c)** Representative images of the cells stained with an anti-SF3b1 primary antibody and an Alexa-647-labeled secondary antibody (left) and summary of the anti-SF3b1 foci morphologies (right). Norm._area [%] is the area of an SF3b1 focus normalized by a maximum value for both groups (0.8 μ^2^). Sample size: 60–68 foci for each biological repeat. *p < 0.05; **p < 0.01; ***p < 0.001. Scale bar: 10 μm.

Fluorescence microscopy images of HEK-293 cells transfected with FAM-C_5_T^propyl^-TAMRA (Figure 2a) were obtained after incubating them for 30 min in either native or acidic (pH 6) media. The latter yielded a decreased FAM signal in the nucleus, confirming the validity of the acidosis model. TAMRA quenching was minimal and did not prevent the morphological characterization of speckles where the sensor signal was enriched, consistent with a previous study (Petrunina et al., 2023). The speckle areas were normalized to a maximum value obtained under native conditions. Despite pronounced variance in both groups, statistical analysis revealed a significant decrease of the median speckle size (∼17%) and a noticeable increase of circularity (∼7%) in acidosis compared to native conditions. Similar acidosis-induced changes were obtained in SH-SY5Y neuroblastoma cells (Supplementary Figure S3). These changes were less pronounced than those observed in a simplified *in vitro* model (Figure 1c), but the trends were similar.

Because sensor interference with speckle morphology could not be ruled out, we additionally verified the pH-induced changes using immunofluorescent staining with antibodies (Abs) to the U2 snRNP component SF3B1, which is found at the periphery and within the core of the speckles (Girard et al., 2012), as well as with antibodies to the core speckle components SRRM2 and SRSF2A (Ilik et al., 2020). FITC-labeled anti-SRRM2/SRSF2 Ab was visualized in the green channel (Figure 2b). Its quantification confirmed a significant (approximately 2-fold) decrease in the median speckle area at pH 6. Anti-SF3b1 Ab was stained with Alexa Fluor 647-labeled secondary Abs and visualized in the red channel (Figure 2c). Its quantificantion confirmed a significant (approximately 1.4-fold) increase in the median circularity. We conclude that acute acidosis reshapes nuclear speckles in a manner consistent with sequestering SRSFs and probably other speckle components. Such redistribution of splicing factors is likely to alter splice site selection. However, the accumulation of alternatively spliced transcripts may require several hours, which is incompatible with the acute acidosis model because prolonged incubation at pH 6 decreases cell viability (Dubourg et al., 2023). Therefore, we switched to a previously reported model of non-damaging (mild) chronic acidosis (Dubourg et al., 2023).

### 3.3 Acidification affects alternative splicing, promoting SRSF-sensitive exon skipping

To study the effects of chronic acidosis, HEK-293 cells were incubated in normal or mildly acidified (pH 6.6) media (Dubourg et al., 2023) for 2 days. In parallel, we incubated the cells with a selective SR protein kinase inhibitor, SRPIN340 (Fukuhara et al., 2006) in normal-pH media at a subtoxic concentration of 10 μM (He et al., 2022) to compare acidosis to SRSF dephosphorylation. After 2 days, total RNA was extracted and used for poly(A)-selected RNA sequencing, and the coding transcripts were analyzed. Principal component analysis (PCA) confirmed that the samples treated with acidified media separated clearly from the control samples and those treated with SRPIN340 (Supplementary Figure S6a). The selective AS regulator SRPIN340 had minimal effects on transcription, whereas acidosis induced noticeable transcriptional reprogramming (Supplementary Figure S6b and Supplementary Data Sheet 3).

Interferon signaling and fatty acid metabolism, which are commonly dysregulated in acidic cancer tissues (Corbet et al., 2016), were among the top enriched categories revealed by the functional analysis of all genes downregulated by acidosis (Supplementary Figure S7a and Supplementary Data Sheet 4). Among the upregulated genes, those related to cellular senescence were particularly enriched (Supplementary Figure S7b). These findings are also consistent with previous reports (Böhme and Bosserhoff, 2020; Chiheb et al., 2025). The senescent-like phenotype was accompanied by the downregulation of DNA repair-related genes and altered splicing of their transcripts (Supplementary Figure S8a), as well as the upregulation of TP53-associated regulatory genes and altered splicing of their transcripts (Supplementary Figure S8b).

**FIGURE 3:**
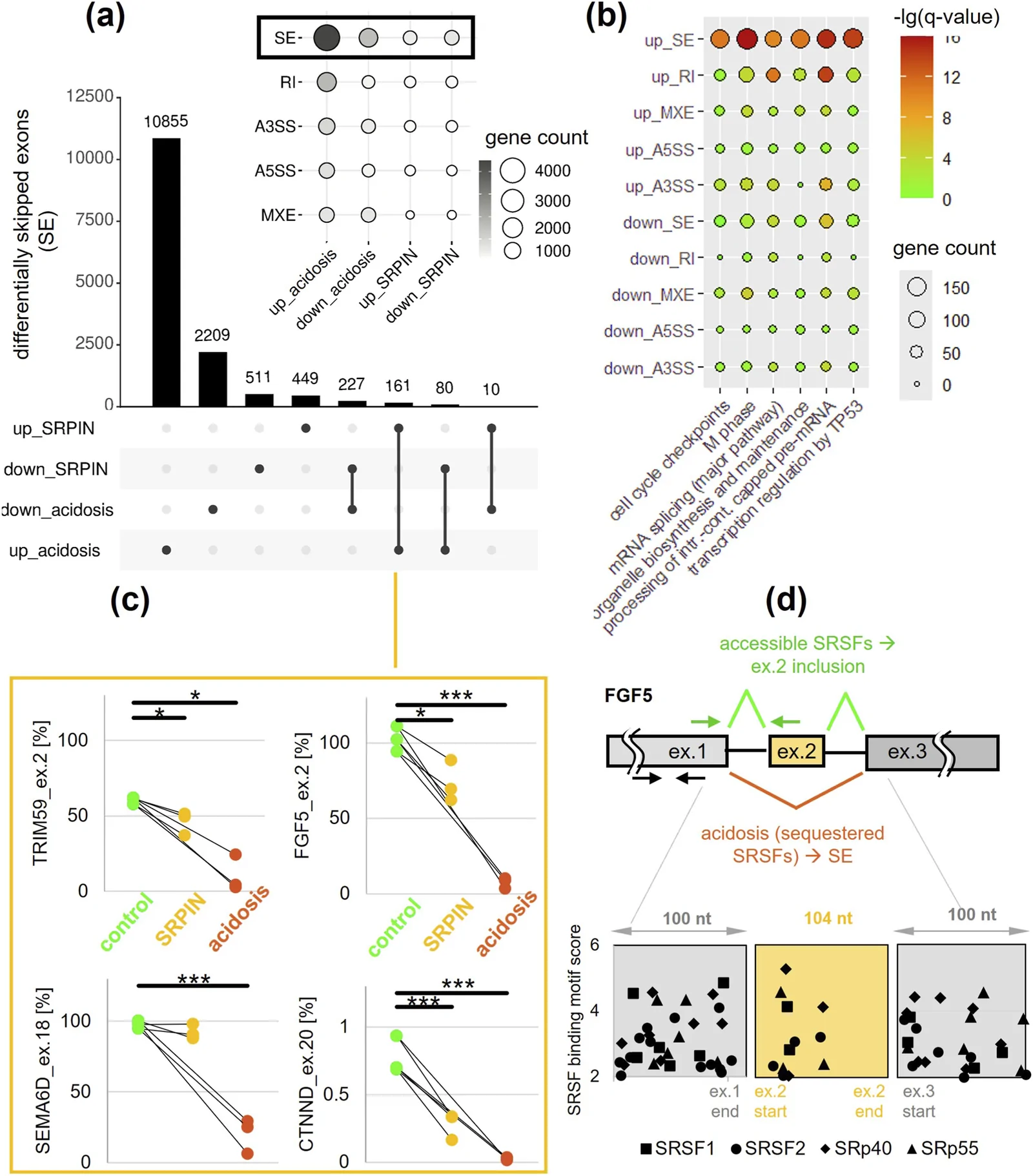
Alternative splicing (AS) changes in HEK-293T cells induced by acidosis and SRSF kinase inhibitor SRPIN340. **(a)** Relative frequencies of differential AS events: numbers of genes with regulated AS events and significance or regulation (dot plot) and detailed analysis of differential exon skipping (UpSet plot). Up(down) _acidosis/SRPIN indicates increased (decreased) frequency of a particular AS event under specified conditions compared to control (treatment with a blank sample). SE, skipped exon; RI, retained intro; A5SS, alternative 5′ donor sites; A3SS, alternative 3′ acceptor sites; MXE, mutually exclusive exons. The black box in the dot plot indicates a subset of AS events used for the UpSet plot. **(b)** Functional analysis of genes whose transcripts show increased (up_) or decreased (down_) AS frequencies: the most significant enriched REACTOME terms. Inclusion criterion: −log10 (q-value) ≥10 for the most common differential AS event (up_SE, skipped exons under acidosis). **(c)** Verification of up_SE events for selected transcripts. Inclusion of the specified exons [%] under different conditions was calculated based on RT-PCR results with primers to reference adjacent exons and skipped exon-reference exon junctions. Significant condition-dependence of exon inclusion/skipping is marked: *p < 0.05, ***p < 0.001. **(d)** Top: schematic representation of the FGF5 transcript, its splicing under normal conditions (green) and acidosis (orange). Black and green arrows indicate pairs of primers to the reference exons (ex.1) and the ex.1−ex.2 junction, which were used to evaluate ex.2 inclusion percentage. Bottom: the distribution of predicted SRSF binding motifs in FGF5 exon 2 and the flanking regions of adjacent exons. Motifs that are significantly different from the consensus ones (score below the threshold value of 2) are not shown.

A detailed analysis of the alternative splicing (AS) landscape (Figure 3) revealed partial overlap between acidosis- and SRPIN340-induced AS changes (Supplementary Figure S9). Consistent with the presumed interference of SRPIN340 and acidosis with SRSF binding to exonic splicing enhancers, exon skipping was the most frequent AS event regulated by both factors (Figure 3a). Skipped exons (SE) accounted for 61% of all AS events affected by acidosis (50% upregulated and 11% downregulated) and 54% of all AS events affected by SRPIN (23% upregulated and 31% downregulated). The respective numbers of affected genes were 6086 (4492 up_acidosis and 1594 down_acidosis) and 1098 (476 up_SRPIN and 622 down_SRPIN). Retained introns (RI) were the second most common AS events regulated by acidosis, accounting for 16% of all affected AS events (14% up_acidosis and 2% down_acidosis) and 2059 affected genes. The contributions of mutually exclusive exons (MXE) or alternative donor/acceptor sites (A5SS/A3SS) were minor to moderate, accounting for 7%–9% of all AS events (615–1511 genes) affected by acidosis and 4%–16% events (91–387 genes) affected by SRPIN (Figure 3a). It should be noted that some AS events, including RI, may be underestimated in our analysis, because the conventional transcriptome was used as a reference. This is a limitation of our study.

A functional analysis of genes whose transcripts exhibited an increased SE frequency in response to acidosis revealed several terms that were also significantly enriched for other AS events (Figure 3b). These terms can be divided into two categories: (i) checkpoint/proliferation-related terms (including M phase), and (ii) pre-mRNA processing-related terms (including the major splicing pathway). The first category resembles gene sets that reportedly respond to splicing factor knockdowns (Ivanova et al., 2023). The second category aligns with the cellular component analysis, specifically the enrichment of speckle component-encoding transcripts among acidosis- and SRPIN340-sensitive transcripts (Supplementary Figure S9). This suggests potential secondary effects on splicing.

The key primary effect—exon skipping, presumably due to SRSF sequestration from exonic splicing enhancers—was verified for selected SEs from the subset of acidosis- and SRPIN340-sensitive transcripts (Figure 3a). The exon inclusion percentage (Figure 3d) was calculated based on the relative cDNA levels evaluated by quantitative RT-PCR (Supplementary Figure S10) using primers for reference exons and skipped/reference exon junctions (Supplementary Table S1). The selected transcripts encoded E3 ligase TRIM59, fibroblast growth factor FGF5, semaphorin SEMA6D, and catenin CTNND1. These regulators of cell proliferation, apoptosis, and migration have previously been considered as prognostic cancer markers (Schackmann et al., 2013; Huang et al., 2018; Huang et al., 2022; Chiang and Yap, 2024; Shi et al., 2026). A schematic representation of verified AS events with marked SEs in *TRIM59*, *SEMA6D*, and *CTNND1* transcripts is shown in Supplementary Figure S11; FGF5 is shown in Figure 3d. For *SEMA6D*, RT-PCR confirmed a significant decrease in the inclusion of the exons of interest in response to acidosis. For *TRIM59*, *CTNND1*, and *FGF5* the effects of both SRPIN340 and acidosis were significant (p < 0.05).

We next questioned whether the confirmed SEs differ from constitutively spliced exons in terms of the SRSF binding capacity. Using the exonic splicing enhancer (ESE) search tool (Cartegni, 2003), we mapped sequences matching the consensus binding motifs of four well-characterized SRSFs (ESE motif similarity score >2) and compared their frequencies in SEs and the adjacent constitutively spliced (reference) exons. In the case of *FGF5*, the SE was clearly depleted of SRSF binding motifs compared to both downstream and upstream exons (Figure 3d). For *CTNND1*, *TRIM59*, and *SEMA6D* (Supplementary Figure S11), no trend was apparent. However, the analysis of all 161 SEs that were skipped in response to both acidosis and SRPIN340 (Figure 3a) revealed a lower median frequency of SRSF binding motifs per 100 nt compared to the frequencies in the upstream and downstream exons (Supplementary Figure S12). This difference may account for the different exon sensitivity to SRSF accessibility/sequestration.

Finally, we partially verified one possible alternative pathway behind the observed AS changes: the secondary effects of altered splicing factor gene expression. Previously, profound changes in the AS landscape were described for late-stage neuroblastoma (Guo et al., 2011), and the dysregulation of *MYCN*-dependent small nuclear ribonuclear polypeptides (SNRPs), which are spliceosome subunits, was found to be among the key drivers of these changes (Salib et al., 2024). Our RNA-seq data indicated altered levels of SNRP A, B, D2, and D3 in the case of acidosis (Supplementary Data Sheet 3). Based on previous reports (Fan et al., 2024; Salib et al., 2024; Li et al., 2025), increased SE and RI frequency could be attributed to *SNRPD* repression, while overexpression of *SNRPD*s is associated with normal splicing. We observed minor-to-moderate *SNRPD* upregulation in acidosis and confirmed it for *SNRPD3* by quantitative RT-PCR (Supplementary Figure S12). Consistent with the *SNRPD* upregulation, its negative regulator *MYCN* was slightly repressed in acidosis (Supplementary Figure S10). Thus, although the core spliceosomal subunits were affected, their altered transcription does not explain the major AS changes.

In summary, we showed that acidosis alters the AS landscape, affecting the transcripts of cell cycle- and proliferation-related genes, as well as splicing-related genes. The most commonly affected AS type, SE, supports the SRSF-dependent mechanism. The increased frequency of SE in acidosis is consistent with the presumed SRSF sequestration in speckles.

## 4 Discussion

Pathological transcriptome-wide AS changes are typically attributed to the dysregulation of either SRSFs or hnRNPs (Tao et al., 2024). Previously described common causes of such dysregulation include mutations in SRSF/hnRNP-encoding genes in leukemia (Lee et al., 2018), overexpression of these genes or aberrant posttranscriptional modulation by microRNAs in lung cancer (Coomer et al., 2019) and breast cancer (Yang et al., 2019) and malfunction of SRSF/hnRNP-modifying enzymes. Acidosis is another likely cause (Cui et al., 2024). Unlike the aforementioned causes, acidosis induces multi-path perturbations of the cellular metabolism, which makes interpreting AS changes particularly challenging. Here, we partially verified the hypothesis of SRSF-dependent AS dysregulation in acidosis. Potential indirect pathways remained mostly outside the scope of the study, which is its key limitation. We presented evidence that moderate acidosis, characteristic of the tumor microenvironment, alters the accessibility of SRSFs and hnRNPs (primarily SRSFs) by sequestering them in nuclear speckles and thus induces AS reprogramming directly. This mechanistic aspect of AS regulation may have been underestimated in previous studies. Only recently have alterations in speckle morphology, compositional signatures, and localization come to light as hallmarks of AS dysregulation and prognostic markers in cancer (Alexander et al., 2025).

We demonstrated that acidosis transforms the typically loose, irregularly shaped nuclear speckles in HEK-293 cells and SH-SY5Y cells into smaller, circular foci (Figure 2). We interpreted this transformation as SRSF-driven solidification of the speckles. The increased frequency of exon skipping (SE) in acidified HEK-293 cells (Figure 3) aligns with this interpretation. However, it should be noted that SE is the most common AS type in both normal and cancer cells even under native conditions (Black, 2003; Kahles et al., 2018). Our assumption about the primary role of SRSFs rather than hnRNPs in the acidosis-driven solidification of the speckles was based on the previously proposed scheme (Cui et al., 2024) and fact that only SRSFs contain clusters of protonation-prone residues (S^p^). We supported this assumption by comparing the sensitivity of phospho-SR and hnRNP A1 to acidification using FRAP assays with model speckles *in vitro*. These assays revealed a significant reduction of phospho-SR mobility but only a slight reduction of hnRNP A1 mobility (Figure 1). An important limitation of these assays is the simplified content of the reconstructed speckles compared to native ones. In many ways, synthetic triple-phosphorylated and dephosphorylated SR peptides do not replicate native SRSFs with multiple phosphorylation sites. However, they enabled direct verification of the impact of S^p^ protonation on phase separation, at least in model systems.

Our thesis about the key role S^p^ residues was supported by the similarities between the effects of acidification and dephosphorylation (Figure 1). In terms of charge distribution, partial S^p^ protonation in acidic media is analogous to partial dephosphorylation. The latter reportedly promotes the formation of denser droplets in LLPS-prone SRSF-RNA mixtures and SRSF aggregation in the absence of RNA (Kundinger et al., 2021). LLPS supposedly complies with a sticker-and-spacer model, in which arginine residues capable of ionic or cation-*pi* interactions with the RNA backbone or nucleobases act as stickers, and non-phosphorylated serine residues act as spacers (Mittag and Pappu, 2022). Unlike dephosphorylated and protonated SRSFs, phosphorylated SRSFs can form transient hairpins through homotypic ionic interactions and undergo RNA-independent LLPS (Yamazaki et al., 2022) at the core of the speckles due to their pronounced blockiness of charge (BLC). This renders speckles flexible and loose (Zhang et al., 2025).

In cells, the effects of phosphorylation regulation are less straightforward than the effects of pH alteration because kinase dependence varies across the SRSF family. For example, the hotspots of SRPK-mediated phosphorylation in SRSF1 are clustered in the LCR N-terminus, while in SRSF3 they are randomly dispersed, resulting in pronounced and minor BLC, respectively, and slightly different LLPS preferences (Long et al., 2019). This explains the existence of speckle subdomains (Tripathi et al., 2012; Ilik et al., 2020; Zhang et al., 2024; Małszycki et al., 2026) and their dynamic behavior, which is governed by SRPK, CKL, and CK2 (Wang et al., 2024; Lázaro et al., 2025; Aubol et al., 2018; Gui et al., 1994; Kuroyanagi et al., 1998) and must be sensitive to both pH and kinase inhibitors. Here, we compared acidosis to the SRPK inhibitor SRPIN340, which induces the solidification and eventual elimination of speckles form the nucleus (Kundinger et al., 2021). We observed only partial overlap between the effects of SRPIN340 and acidosis on AS. Exon skipping was generally inhibited by SRPIN340, which can be attributed to compensatory effects, underscoring the complexity of the kinase network. Furthermore, compensatory effects may be cell line-specific. We only reported transcriptome-wise AS analysis for HEK-293 cells. This is an additional important limitation of our study, and further investigation in this field is needed to verify the universality of the discussed mechanism.

Thus, in addition to elucidating the pathological impact of acidosis and highlighting its possible contribution to cancer progression, our findings encourage future studies on the interplay between acidosis with anticancer candidates based on SRPK, CKL, or CK2 kinase inhibitors (Kim et al., 2014), which may partially compensate for or exacerbate AS reprogramming.

## Data Availability

The datasets generated for this study can be found in the Gene Expression Omnibus (GEO) repository (https://www.ncbi.nlm.nih.gov/geo/) under the accession number GSE328432.

## Supplementary material

SUPPLEMENTARY DATA SHEET 1 Droplet fusion.

SUPPLEMENTARY DATA SHEET 2 Microsphere movement inside the droplets.

SUPPLEMENTARY DATA SHEET 3 Changes in gene expression and alternative splicing.

SUPPLEMENTARY DATA SHEET 4 Functional analysis of affected genes.

## References

[B1] Alexander K. A. Yu R. Skuli N. Coffey N. J. Nguyen S. Faunce C. L. (2025). Nuclear speckles regulate functional programs in cancer. Nat. Cell Biol. 27, 322–335. 10.1038/s41556-024-01570-0 PMC12039181 39747580

[B2] Alshareedah I. Kaur T. Banerjee P. R. (2021). Methods for characterizing the material properties of biomolecular condensates. Methods Enzymol. 646, 143–183. 10.1016/BS.MIE.2020.06.009 33453924 PMC7849318

[B3] Aubol B. E. Keshwani M. M. Fattet L. Adams J. A. (2018). Mobilization of a splicing factor through a nuclear kinase–kinase complex. Biochem. J. 475, 677–690. 10.1042/BCJ20170672 29335301 PMC6293969

[B4] Black D. L. (2003). Mechanisms of alternative pre-messenger RNA splicing. Annu. Rev. Biochem. 72, 291–336. 10.1146/annurev.biochem.72.121801.161720 12626338

[B5] Böhme I. Bosserhoff A. (2020). Extracellular acidosis triggers a senescence-like phenotype in human melanoma cells. Pigment. Cell Melanoma Res. 33, 41–51. 10.1111/pcmr.12811 31310445

[B6] Bolger A. M. Lohse M. Usadel B. (2014). Trimmomatic: a flexible trimmer for illumina sequence data. Bioinformatics 30, 2114–2120. 10.1093/bioinformatics/btu170 24695404 PMC4103590

[B7] Bradley R. K. Anczuków O. (2023). RNA splicing dysregulation and the hallmarks of cancer. Nat. Rev. Cancer 23, 135–155. 10.1038/s41568-022-00541-7 PMC10132032 36627445

[B8] Cartegni L. Wang J. Zhu Z. Zhang M. Q. Krainer A. R. (2003). ESEfinder: a web resource to identify exonic splicing enhancers. Nucleic Acids Res. 31, 3568–3571. 10.1093/nar/gkg616 12824367 PMC169022

[B9] Chiang D. C. Yap B. K. (2024). TRIM25, TRIM28 and TRIM59 and their protein partners in cancer signaling crosstalk: potential novel therapeutic targets for cancer. Curr. Issues Mol. Biol. 46, 10745–10761. 10.3390/cimb46100638 PMC11506413 39451518

[B10] Chiheb C. Fischer S. El Ahmad Z. Henz I. Böhme-Schäfer I. Kappelmann-Fenzl M. (2025). Acidic melanoma microenvironment selects for a senescence-like but also migratory-active subpopulation driving metastatic disease. Cell Death Discov. 11, 469. 10.1038/s41420-025-02806-0 PMC12537852 41115919

[B11] Coomer A. O. Black F. Greystoke A. Munkley J. Elliott D. J. (2019). Alternative splicing in lung cancer. Biochimica Biophysica Acta (BBA) - Gene Regul. Mech. 1862, 194388. 10.1016/j.bbagrm.2019.05.006 31152916

[B12] Corbet C. Feron O. (2017). Tumour acidosis: from the passenger to the driver’s seat. Nat. Rev. Cancer 17, 577–593. 10.1038/nrc.2017.77 28912578

[B13] Corbet C. Pinto A. Martherus R. Santiago de Jesus J. P. Polet F. Feron O. (2016). Acidosis drives the reprogramming of fatty acid metabolism in cancer cells through changes in mitochondrial and histone acetylation. Cell Metab. 24, 311–323. 10.1016/j.cmet.2016.07.003 27508876

[B14] Cui H. Shi Q. Macarios C. M. Schimmel P. (2024). Metabolic regulation of mRNA splicing. Trends Cell Biol. 34, 756–770. 10.1016/j.tcb.2024.02.002 38431493

[B15] Damaghi M. Wojtkowiak J. W. Gillies R. J. (2013). pH sensing and regulation in cancer. Front. Physiol. 4, 370. 10.3389/fphys.2013.00370 24381558 PMC3865727

[B16] Dobin A. Davis C. A. Schlesinger F. Drenkow J. Zaleski C. Jha S. (2013). STAR: ultrafast universal RNA-seq aligner. Bioinformatics 29, 15–21. 10.1093/bioinformatics/bts635 23104886 PMC3530905

[B17] Dubourg V. Schulz M.-C. Terpe P. Ruhs S. Kopf M. Gekle M. (2023). Hypothesis-generating analysis of the impact of non-damaging metabolic acidosis on the transcriptome of different cell types: integrated stress response (ISR) modulation as general transcriptomic reaction to non-respiratory acidic stress? PLoS One 18, e0290373. 10.1371/journal.pone.0290373 37624790 PMC10456223

[B18] Erkelenz S. Mueller W. F. Evans M. S. Busch A. Schöneweis K. Hertel K. J. (2013). Position-dependent splicing activation and repression by SR and hnRNP proteins rely on common mechanisms. RNA 19, 96–102. 10.1261/rna.037044.112 23175589 PMC3527730

[B19] Fan W. Huang J. Tian F. Hong X. Zhu K. Zhan Y. (2024). M ^6^ a-modified SNRPA controls alternative splicing of ERCC1 exon 8 to induce cisplatin resistance in lung adenocarcinoma. Adv. Sci. 11, 2404609. 10.1002/advs.202404609 39555714 PMC11653629

[B20] Fukuhara T. Hosoya T. Shimizu S. Sumi K. Oshiro T. Yoshinaka Y. (2006). Utilization of host SR protein kinases and RNA-splicing machinery during viral replication. Proc. Natl. Acad. Sci. 103, 11329–11333. 10.1073/pnas.0604616103 16840555 PMC1544086

[B21] Galganski L. Urbanek M. O. Krzyzosiak W. J. (2017). Nuclear speckles: molecular organization, biological function and role in disease. Nucleic Acids Res. 45, 10350–10368. 10.1093/nar/gkx759 28977640 PMC5737799

[B22] Generalova A. N. Sizova S. V. Zdobnova T. A. Zarifullina M. M. Artemyev M. V. Baranov A. V. (2011). Submicron polymer particles containing fluorescent semiconductor nanocrystals Cdse/Zns for bioassays. Nanomedicine 6, 195–209. 10.2217/nnm.10.162 21385123

[B23] Geuens T. Bouhy D. Timmerman V. (2016). The hnRNP family: insights into their role in health and disease. Hum. Genet. 135, 851–867. 10.1007/s00439-016-1683-5 27215579 PMC4947485

[B24] Girard C. Will C. L. Peng J. Makarov E. M. Kastner B. Lemm I. (2012). Post-transcriptional spliceosomes are retained in nuclear speckles until splicing completion. Nat. Commun. 3, 994. 10.1038/ncomms1998 22871813

[B25] Gui J.-F. Lane W. S. Fu X.-D. (1994). A serine kinase regulates intracellular localization of splicing factors in the cell cycle. Nature 369, 678–682. 10.1038/369678a0 8208298

[B26] Guo X. Chen Q.-R. Song Y. K. Wei J. S. Khan J. (2011). Exon array analysis reveals neuroblastoma tumors have distinct alternative splicing patterns according to stage and MYCN amplification status. BMC Med. Genomics 4, 35. 10.1186/1755-8794-4-35 21501490 PMC3096898

[B27] He C. Liu B. Wang H.-Y. Wu L. Zhao G. Huang C. (2022). Inhibition of SRPK1, a key splicing regulator, exhibits antitumor and chemotherapeutic-sensitizing effects on extranodal NK/T-cell lymphoma cells. BMC Cancer 22, 1100. 10.1186/s12885-022-10158-6 PMC9609466 36303126

[B28] Huang Y. Yario T. A. Steitz J. A. (2004). A molecular link between SR protein dephosphorylation and mRNA export. Proc. Natl. Acad. Sci. 101, 9666–9670. 10.1073/pnas.0403533101 15210956 PMC470732

[B29] Huang Y. Wang H. Yang Y. (2018). Expression of fibroblast growth factor 5 (FGF5) and its influence on survival of breast cancer patients. Med. Sci. Monit. 24, 3524–3530. 10.12659/MSM.907798 29804124 PMC5998728

[B30] Huang N. Sun X. Li P. liu X. Zhang X. Chen Q. (2022). TRIM family contribute to tumorigenesis, cancer development, and drug resistance. Exp. Hematol. Oncol. 11, 75. 10.1186/s40164-022-00322-w PMC9583506 36261847

[B31] Huelga S. C. Vu A. Q. Arnold J. D. Liang T. Y. Liu P. P. Yan B. Y. (2012). Integrative genome-wide analysis reveals cooperative regulation of alternative splicing by hnRNP proteins. Cell Rep. 1, 167–178. 10.1016/j.celrep.2012.02.001 22574288 PMC3345519

[B32] Ilik İ. A. Malszycki M. Lübke A. K. Schade C. Meierhofer D. Aktaş T. (2020). SON and SRRM2 are essential for nuclear speckle formation. Elife 9, e60579. 10.7554/eLife.60579 33095160 PMC7671692

[B33] Ivanova O. M. Anufrieva K. S. Kazakova A. N. Malyants I. K. Shnaider P. V. Lukina M. M. (2023). Non-canonical functions of spliceosome components in cancer progression. Cell Death Dis. 14, 77. 10.1038/s41419-022-05470-9 PMC9895063 36732501

[B34] Jobbins A. M. Campagne S. Weinmeister R. Lucas C. M. Gosliga A. R. Clery A. (2022). Exon-independent recruitment of SRSF1 is mediated by U1 snRNP stem-loop 3. EMBO J. 41, EMBJ2021107640. 10.15252/embj.2021107640 PMC8724738 34779515

[B35] Kahles A. Lehmann K.-V. Toussaint N. C. Hüser M. Stark S. G. Sachsenberg T. (2018). Comprehensive analysis of alternative splicing across tumors from 8,705 patients. Cancer Cell 34, 211–224.e6. 10.1016/j.ccell.2018.07.001 30078747 PMC9844097

[B36] Kim H. Choi K. Kang H. Lee S.-Y. Chi S.-W. Lee M.-S. (2014). Identification of a novel function of CX-4945 as a splicing regulator. PLoS One 9, e94978. 10.1371/journal.pone.0094978 24743259 PMC3990583

[B37] Korenchan D. E. Flavell R. R. (2019). Spatiotemporal pH heterogeneity as a promoter of cancer progression and therapeutic resistance. Cancers (Basel) 11, 1026. 10.3390/cancers11071026 31330859 PMC6678451

[B38] Kundinger S. R. Dammer E. B. Yin L. Hurst C. Shapley S. Ping L. (2021). Phosphorylation regulates arginine-rich RNA-binding protein solubility and oligomerization. J. Biol. Chem. 297, 101306. 10.1016/j.jbc.2021.101306 PMC8569591 34673031

[B39] Kuroyanagi N. Onogi H. Wakabayashi T. Hagiwara M. (1998). Novel SR-Protein-Specific kinase, SRPK2, disassembles nuclear speckles. Biochem. Biophys. Res. Commun. 242, 357–364. 10.1006/bbrc.1997.7913 9446799

[B40] Lázaro B. Tadeo-Masa F. J. Rodriguez A. Ayuso L. Martínez-Láinez J. M. Quandt E. (2025). Nuclear Speckle Dynamics are Controlled by Polyphosphate Inhibition of CLK Proteins. 10.1101/2025.01.15.633116 PMC13076214 41978265

[B41] Lee S. C.-W. North K. Kim E. Jang E. Obeng E. Lu S. X. (2018). Synthetic lethal and convergent biological effects of cancer-associated spliceosomal gene mutations. Cancer Cell 34, 225–241.e8. 10.1016/j.ccell.2018.07.003 PMC6373472 30107174

[B42] Li D. Yu W. Lai M. (2023). Towards understandings of serine/arginine-rich splicing factors. Acta Pharm. Sin. B 13, 3181–3207. 10.1016/j.apsb.2023.05.022 PMC10465970 37655328

[B43] Li Y. Chen Z. Xiao H. Liu Y. Zhao C. Yang N. (2025). Targeting the splicing factor SNRPB inhibits endometrial cancer progression by retaining the POLD1 intron. Exp. Mol. Med. 57, 420–435. 10.1038/s12276-025-01407-2 PMC11873159 39910288

[B44] Liberzon A. Subramanian A. Pinchback R. Thorvaldsdóttir H. Tamayo P. Mesirov J. P. (2011). Molecular signatures database (MSigDB) 3.0. Bioinformatics 27, 1739–1740. 10.1093/bioinformatics/btr260 21546393 PMC3106198

[B45] Long Y. Sou W. H. Yung K. W. Y. Liu H. Wan S. W. C. Li Q. (2019). Distinct mechanisms govern the phosphorylation of different SR protein splicing factors. J. Biol. Chem. 294, 1312–1327. 10.1074/jbc.RA118.003392 30478176 PMC6349114

[B46] Love M. I. Huber W. Anders S. (2014). Moderated Estimation of Fold Change and Dispersion for RNA-seq Data with DESeq2. 10.1101/002832 PMC4302049 25516281

[B47] Maertens G. N. Cook N. J. Wang W. Hare S. Gupta S. S. Öztop I. (2014). Structural basis for nuclear import of splicing factors by human transportin 3. Proc. Natl. Acad. Sci. U. S. A. 111, 2728–2733. 10.1073/pnas.1320755111 24449914 PMC3932936

[B48] Małszycki M. Martina L. Ilık İ. A. Salgado Figueroa D. Dasgupta N. Çoşar M. F. (2026). Nuclear speckles enable processing of RNA from GC-rich isochores. Cell 189, 2024–2039.e25. 10.1016/j.cell.2026.01.011 41747727 PMC13283296

[B49] Martin E. W. Thomasen F. E. Milkovic N. M. Cuneo M. J. Grace C. R. Nourse A. (2021). Interplay of folded domains and the disordered low-complexity domain in mediating hnRNPA1 phase separation. Nucleic Acids Res. 49, 2931–2945. 10.1093/nar/gkab063 33577679 PMC7969017

[B50] Michlewski G. Sanford J. R. Cáceres J. F. (2008). The splicing factor SF2/ASF regulates translation initiation by enhancing phosphorylation of 4E-BP1. Mol. Cell 30, 179–189. 10.1016/j.molcel.2008.03.013 18439897

[B51] Mittag T. Pappu R. V. (2022). A conceptual framework for understanding phase separation and addressing open questions and challenges. Mol. Cell 82, 2201–2214. 10.1016/j.molcel.2022.05.018 35675815 PMC9233049

[B52] Naro C. Bielli P. Sette C. (2021). Oncogenic dysregulation of pre-mRNA processing by protein kinases: challenges and therapeutic opportunities. FEBS J. 288, 6250–6272. 10.1111/febs.16057 34092037 PMC8596628

[B53] Okajima F. (2013). Regulation of inflammation by extracellular acidification and proton-sensing GPCRs. Cell. Signal. 25, 2263–2271. 10.1016/j.cellsig.2013.07.022 23917207

[B54] Pandit S. Zhou Y. Shiue L. Coutinho-Mansfield G. Li H. Qiu J. (2013). Genome-wide analysis reveals SR protein cooperation and competition in regulated splicing. Mol. Cell 50, 223–235. 10.1016/j.molcel.2013.03.001 23562324 PMC3640356

[B55] Patro R. Duggal G. Love M. I. Irizarry R. A. Kingsford C. (2017). Salmon provides fast and bias-aware quantification of transcript expression. Nat. Methods 14, 417–419. 10.1038/nmeth.4197 28263959 PMC5600148

[B56] Paul S. Arias M. A. Wen L. Liao S. E. Zhang J. Wang X. (2024). RNA molecules display distinctive organization at nuclear speckles. iScience 27, 109603. 10.1016/j.isci.2024.109603 38638569 PMC11024929

[B57] Pearlman S. M. Serber Z. Ferrell J. E. (2011). A mechanism for the evolution of phosphorylation sites. Cell 147, 934–946. 10.1016/j.cell.2011.08.052 22078888 PMC3220604

[B58] Petrunina N. A. Shtork A. S. Lukina M. M. Tsvetkov V. B. Khodarovich Y. M. Feofanov A. V. (2023). Ratiometric i-Motif-Based sensor for precise long-term monitoring of pH micro alterations in the nucleoplasm and interchromatin granules. ACS Sens. 8, 619–629. 10.1021/acssensors.2c01813 36662613

[B59] Pillai S. R. Damaghi M. Marunaka Y. Spugnini E. P. Fais S. Gillies R. J. (2019). Causes, consequences, and therapy of tumors acidosis. Cancer Metastasis Rev. 38, 205–222. 10.1007/s10555-019-09792-7 30911978 PMC6625890

[B60] Saha K. Ghosh G. (2022). Cooperative engagement and subsequent selective displacement of SR proteins define the pre-mRNA 3D structural scaffold for early spliceosome assembly. Nucleic Acids Res. 50, 8262–8278. 10.1093/nar/gkac636 35871302 PMC9371905

[B61] Salib A. Jayatilleke N. Seneviratne J. A. Mayoh C. De Preter K. Speleman F. (2024). MYCN and SNRPD3 cooperate to maintain a balance of alternative splicing events that drives neuroblastoma progression. Oncogene 43, 363–377. 10.1038/s41388-023-02897-y 38049564 PMC10824661

[B62] Schackmann R. C. J. Tenhagen M. van de Ven R. A. H. Derksen P. W. B. (2013). p120-catenin in cancer – mechanisms, models and opportunities for intervention. J. Cell Sci. 126, 3515–3525. 10.1242/jcs.134411 23950111

[B63] Shen S. Park J. W. Lu Z. Lin L. Henry M. D. Wu Y. N. (2014). rMATS: robust and flexible detection of differential alternative splicing from replicate RNA-seq data. Proc. Natl. Acad. Sci. 111, E5593–E5601. 10.1073/pnas.1419161111 25480548 PMC4280593

[B64] Shi W. Zhang F. Sun W.-Q. Liang L.-Q. Krishnan S. (2026). Semaphorin 6D drives anti-tumor type I interferon responses to reprogram the tumor microenvironment in colorectal cancer. Cell Death Dis. 17, 256. 10.1038/s41419-026-08478-7 PMC12979794 41735257

[B65] Shtork A. S. Pavlova Iu. I. Svetlova J. I. Iudin M. S. Grafskaia E. N. Manuvera V. A. (2025). Quantitative analysis of biomolecular condensates on a modified support. Russ. J. Bioorg. Chem. 51, 273–284. 10.1134/S1068162025010182

[B66] Śmiechowski M. (2010). Theoretical pKa prediction of O-phosphoserine in aqueous solution. Chem. Phys. Lett. 501, 123–129. 10.1016/j.cplett.2010.10.063

[B67] Soneson C. Love M. I. Robinson M. D. (2016). Differential analyses for RNA-seq: transcript-level estimates improve gene-level inferences. F1000Res 4, 1521. 10.12688/f1000research.7563.2 26925227 PMC4712774

[B68] Song M. Pang L. Zhang M. Qu Y. Laster K. V. Dong Z. (2023). Cdc2-like kinases: structure, biological function and therapeutic targets for diseases. Signal Transduct. Target. Ther. 8, 148. 10.1038/s41392-023-01409-4 37029108 PMC10082069

[B69] Tao Y. Zhang Q. Wang H. Yang X. Mu H. (2024). Alternative splicing and related RNA binding proteins in human health and disease. Signal Transduct. Target. Ther. 9, 26. 10.1038/s41392-024-01734-2 PMC10835012 38302461

[B70] Tripathi V. Ellis J. D. Shen Z. Song D. Y. Pan Q. Watt A. T. (2010). The nuclear-retained noncoding RNA MALAT1 regulates alternative splicing by modulating SR splicing factor phosphorylation. Mol. Cell 39, 925–938. 10.1016/j.molcel.2010.08.011 20797886 PMC4158944

[B71] Tripathi V. Song D. Y. Zong X. Shevtsov S. P. Hearn S. Fu X.-D. (2012). SRSF1 regulates the assembly of pre-mRNA processing factors in nuclear speckles. Mol. Biol. Cell 23, 3694–3706. 10.1091/mbc.e12-03-0206 22855529 PMC3442416

[B72] Turaev A. V. Isaakova E. A. Severov V. V. Bogomazova A. N. Zatsepin T. S. Sardushkin M. V. (2021). Genomic DNA i-motifs as fast sensors responsive to near-physiological pH microchanges. Biosens. Bioelectron. 175, 112864. 10.1016/j.bios.2020.112864 33309217

[B73] Vassilev L. T. (2006). Cell cycle synchronization at the G _2_/M phase border by reversible inhibition of CDK1. Cell Cycle 5, 2555–2556. 10.4161/cc.5.22.3463 17172841

[B74] Vedekhina T. Svetlova J. Pavlova I. Barinov N. Alieva S. Malakhova E. (2024). Cross-effects in folding and phase transitions of hnRNP A1 and C9Orf72 RNA G4 *in vitro* . Molecules 29, 4369. 10.3390/molecules29184369 PMC11434081 39339364

[B75] Wang B. Li J. Song Y. Qin X. Lu X. Huang W. (2024). CLK2 condensates reorganize nuclear speckles and induce intron retention. Adv. Sci. 11, 2309588. 10.1002/advs.202309588 PMC11481226 39119950

[B76] Yamazaki H. Takagi M. Kosako H. Hirano T. Yoshimura S. H. (2022). Cell cycle-specific phase separation regulated by protein charge blockiness. Nat. Cell Biol. 24, 625–632. 10.1038/s41556-022-00903-1 35513709 PMC9106583

[B77] Yang Q. Zhao J. Zhang W. Chen D. Wang Y. (2019). Aberrant alternative splicing in breast cancer. J. Mol. Cell Biol. 11, 920–929. 10.1093/jmcb/mjz033 31065692 PMC6884705

[B78] Yu G. He Q.-Y. (2015). ReactomePA: an R/Bioconductor package for reactome pathway analysis and visualization. Mol. Biosyst. 12, 477–479. 10.1039/C5MB00663E 26661513

[B79] Yu G. Wang L.-G. Han Y. He Q.-Y. (2012). clusterProfiler: an R package for comparing biological themes among gene clusters. OMICS 16, 284–287. 10.1089/omi.2011.0118 22455463 PMC3339379

[B80] Zhang M. Gu Z. Guo S. Sun Y. Ma S. Yang S. (2024). SRRM2 phase separation drives assembly of nuclear speckle subcompartments. Cell Rep. 43, 113827. 10.1016/j.celrep.2024.113827 38381607

[B81] Zhang M. Gu Z. Sun Y. Dong Y. Chen J. Shu L. (2025). Phosphorylation-dependent charge blocks regulate the relaxation of nuclear speckle networks. Mol. Cell 85, 1760–1774.e7. 10.1016/j.molcel.2025.03.016 40233760

[B82] Zhou Z. Fu X.-D. (2013). Regulation of splicing by SR proteins and SR protein-specific kinases. Chromosoma 122, 191–207. 10.1007/s00412-013-0407-z 23525660 PMC3660409

[B83] Zhou H. Di Palma S. Preisinger C. Peng M. Polat A. N. Heck A. J. R. (2013). Toward a comprehensive characterization of a human cancer cell phosphoproteome. J. Proteome Res. 12, 260–271. 10.1021/pr300630k 23186163

