# Supplementary material for: Acidosis promotes exon skipping through sequestering SR-rich splicing factors in nuclear speckles
Source: Front Mol Biosci. 2026 Sep 11;13:1862962. doi: 10.3389/fmolb.2026.1862962 (PMC13612490; doi:10.3389/fmolb.2026.1862962)
Supplement: Supplementary file 1 [file Supplementaryfile1.docx]

Supplementary Material

**Figure S1.** Microscopy imaging of phase separation in SR-RNA mixtures. Control images of RNA-free SR samples are shown in the right panel. Conditions: 1 mg/mL dephospho/phospho/mutant-SR(ext) and 1 mg/mL RNA in a 20 mM sodium phosphate (pH 7.4 or 6.0) or 20 mM sodium acetate (pH 4) buffer supplemented with 10% PEG. Scale bar: 20 μm.

**Figure S2.** Microscopy imaging of SR-RNA droplets undergoing fusion on tween-coated glass. Conditions: 1 mg/mL dephospho-SR and 1 mg/mL RNA in a 20 mM sodium phosphate buffer (pH 7.4) supplemented with 10% PEG.

**Figure S3**. Fluorescence microscopy imaging of the co-separation of labeled phospho-SR (green) and hnRNP A1 (red). Top: representative large-filed images (left pane, pH 7.2; right panel, pH 6.0; scale bar: 20 μm) and fluorescence recovery after photobleaching of selected droplets at pH 6 (middle panel, time points: 1, pre-bleach; 2, 0 sec. after bleaching; 3, 40 sec.; 4, 110 sec). In the large-field images, the droplets selected for bleaching and signal normalization are in orange and grey boxes, respectively. Scale bar: 20 μm. Conditions: 1 mg/mL phospho-SR (5% FITC-labeled), 6 μM hnRNP A1 (5% RED-labeled), 3 mg/mL RNA in 20 mM sodium phosphate buffer supplemented with 140 mM KCl. Bottom: summary of droplet morphologies in buffers of different pH. Two independent experiments (biological repeats) with different pH values were performed, each in 3 replicates, and the large-filed images were combined to give the following sample size: N = 228 (bio-rep.1) or 339 (bio-rep.2) for pH 7.2 and N = 694 (bio-rep.1) or 1317 (bio-rep.2) for pH 6.0. The non-parametric Kruskal-Wallis test with post-hoc Dunn’s test revealed no significant difference between the biological repeats (violin plots), so they were combined in scatterplots. Norm._area [%] is the area of a droplet projection normalized by a maximum value for both samples (28 μ^2^). *** p < 0.001.

**Figure S4**. Single particle tracking within the phospho-SR-based droplets. (a) Characterization of the poly(acrolein-co-styrene) microspheres loaded with quantum dots (QDs) by dynamic light scattering: the typical correlation function (left) and size distribution summary (right). (b) Representative confocal microscopy images of droplets with microspheres. In the enlarged images of multiple (left) and single (right) microspheres, the scale bar is 1 μm. The droplets were assembled from 1 mg/mL phospho-SR, 6 μM hnRNP A1, and 3 mg/mL RNA in 20 mM sodium phosphate buffer supplemented with 10% PEG. The dashed white lines mark multiple spheres. (c) Summary of the microsphere tracking results (150 trajectories for each pH). Experimental MSD values (mean ± SE) are plotted as a function of lag time and overlaid with linear fitting.

**Figure S5**. Fluorescence microscopy imaging of **SH-SY5Y** cells transfected with the nucleotropic ratiometric pH sensor FAM-C5Tpropyl-TAMRA. Top: representative images of the G2-synchronized transfected cells after a 30 min incubation in normal media (native pH) or acidified media (pH 6) with ionophores (acidosis). Bottom: summary of TAMRA foci morphologies in cells incubated in native *vs* acidified media. Two independent experiments (biological repeats) with different media were performed, each in 2-3 replicates; the replicates were combined for threshold-based foci selection to give at least 50 foci in each repeat. Occasional foci outside the nuclei (no DAPI signal) observed in the pH 6 samples were excluded manually from the analysis after visual screening. The non-parametric Kruskal-Wallis test with post-hoc Dunn’s test revealed no significant difference between the biological repeats (violin plots), so they were combined in the scatterplots (final sample size in each group: N = 99-102). Norm._area [%] is foci area normalized by a maximum value for both groups (0.8 μ^2^). *p < 0.05; **p < 0.01

**Figure S6**. Regulation of transcription in HEK-293 cells by acidification and treatment with SRPIN340. (a) Principal component analysis of transcriptomic data. (b) Summary of differentially expressed genes that satisfy the following criteria: 1) log_2_(Fold_change) >1 for upregulated genes or log_2_(Fold_change) < -1 for downregulated genes; 2) q-value (FDR-adjusted p-value) < 0.05; 3) TPM > 6.

**Figure S7**. Functional analysis of genes that are downregulated (a) or upregulated (b) by acidosis: top 10 enriched categories. Inclusion criteria: TPM > 6, log_2_FC > 4 or <-4, and FDR < 0.05. Database: REACTOME

**Figure S8**. Functional analysis of genes that are downregulated (a) or upregulated (b) by acidosis and show altered SE/RI frequency upon acidosis: top 10 enriched categories. Inclusion criteria: TPM > 6, log2FC > 4 or <-4, and FDR < 0.05 Database: REACTOME

**Figure S9**. Functional analysis of genes that show common AS changes in response to acidosis and SRPIN340: top ten enriched categories from Gene Ontology cellular compartment database.

**Figure S10**. RT-PCR-based verification of the regulation of selected genes by acidosis and SRPIN340. Relative expression was normalized to that of *TBP.* *p < 0.01.

**Figure S11**. Alternative splicing of selected transcripts confirmed by RT-PCR and the distribution of SRSF binding motifs in the differentially skipped exons. Yellow boxes, acidosis-sensitive differentially skipped exons; grey boxes, upstream/downstream acidosis-insensitive exons; orange lines, splicing in acidosis; green lines, splicing under normal conditions; black arrows, control primers to acidosis-insensitive exons; green arrows, primers to acidosis-sensitive exon junctions.

**Figure S12**. Frequency of SRSF binding motifs in skipped exons compared to upstream and downstream ones. Yellow, exons that are skipped in response to acidification and SRPIN340 treatment (161 differentially skipped exons, i.e., the overlap of the “up_SRPIN” and “up acidosis” categories in Fig. 3a and archive S1); Grey, upstream and downstream exons. Statistical analysis: Wilcox test.

**Table S1.** Primers used for qRT-PCR.

| gene | forward | reverse |
| --- | --- | --- |
| *TBP* | TGTATCCACAGTGAATCTTGGTTG | GGTTCGTGGCTCTCTTATCCTC |
| *HPRT1* | TGACACTGGCAAAACAATGCA | TCCTTTTCACCAGCAAGCT |
| *GAPDH* | AATCCCATCACCATCTTCCA | TGGACTCCACGACGTACTCA |
| *SNRPD3* | TGGCAGCAAAATCCGCTTTC | ACGTCCCATTCCACGTCCTC |
| *MYCN* | GCCACATAAGGGGTTTGCCA | CCGCAAAAGCCACCTCTCAT |
| *FGF5 (exon 1)* | GCTGCCACTGATAGGAACCC | CCACTGGAAACTGCTCTGCT |
| *FGF5 (ex.1-2)* | CCACGAAGCCAATATGTTAAGTGT | TCCTCGTATTCCTACAATCCCCT |
| *TRIM59 (exon 1)* | GGCTCTCTGTGATGTTGGCA | ACGGGTTGAACCTCAGGAAG |
| *TRIM59 (ex.1-2)* | CCAAACCACGAGATCAACAAAGAAA | GAGCATGGCAGTACACGAGG |
| *SEMA6D (exon 19)* | TTTTCCGTCTAGTCCGCCAC | GTTCTGGTGTGCCATCTGGA |
| *SEMA6D (ex. 18-19)* | CCACAATGGCAAGTATCCCAGA | TTCCCATCGTACACCCTTTGG |
| *CTNND1 (exon 21)* | GTCTGAAACTGGCTTCCCCA | GTTTGCCAACCAATCCAGGG |
| *CTNND1 (ex.20-21)* | TGTCTTACCCCTCCATGCAGAA | TGGCACAATAGTCCAGCGAA |
